# *Bacillus amyloliquefaciens* SN16-1-Induced Resistance System of the Tomato against *Rhizoctonia solani*

**DOI:** 10.3390/pathogens11010035

**Published:** 2021-12-29

**Authors:** Huihui Zhao, Xiaobing Wang, Wei Wang

**Affiliations:** State Key Laboratory of Bioreactor Engineering, East China University of Science and Technology, Shanghai 200237, China; huizhao1123@163.com

**Keywords:** biocontrol, plant defense, beneficial bacteria, RAN-Seq, plant–microbe interaction

## Abstract

Tomato (*Solanum lycopersicum*), as an important economical vegetable, is often infected with *Rhizoctonia solani*, which results in a substantial reduction in production. Therefore, the molecular mechanism of biocontrol microorganisms assisting tomato to resist pathogens is worth exploring. Here, we use *Bacillus amyloliquefaciens* SN16-1 as biocontrol bacteria, and employed RNA-Seq technology to study tomato gene and defense-signaling pathways expression. Gene Ontology (GO) analyses showed that an oxidation-reduction process, peptidase regulator activity, and oxidoreductase activity were predominant. Kyoto Encyclopedia of Genes and Genomes (KEGG) analyses showed that phenylpropanoid biosynthesis, biosynthesis of unsaturated fatty acids, aldosterone synthesis and secretion, and phototransduction were significantly enriched. SN16-1 activated defenses in the tomato via systemic-acquired resistance (which depends on the salicylic acid signaling pathway), rather than classic induction of systemic resistance. The genes induced by SN16-1 included transcription factors, plant hormones (ethylene, auxin, abscisic acid, and gibberellin), receptor-like kinases, heat shock proteins, and defense proteins. SN16-1 rarely activated pathogenesis-related proteins, but most pathogenesis-related proteins were induced in the presence of the pathogens. In addition, the molecular mechanisms of the response of tomatoes to SN16-1 and *R. solani* RS520 were significantly different.

## 1. Introduction

Tomato (*Solanum lycopersicum*) is an economically important vegetable throughout the world, with significant importance for human health and nutrition. Tomato damping off, mainly caused by *Rhizoctonia solani*, is a common disease that results in a substantial reduction in production [1]. Chemical methods are used to control the disease, but such pesticides leave residues and may produce adverse effects on the environment and human health [2]. Effective means that accord with the sustainable development of agriculture are being sought to manage this disease. Biological control is a promising alternative to the use of chemicals to protect the plants from the pathogen [3,4].

*Bacillus amyloliquefaciens* is a biological control agent that can control damping off [5,6]. This bacterium possesses a wide variety of mechanisms that compete with pathogens for nutrients and space, produce antifungal antibiotics, inhibit biofilm formation, and induce systemic resistance [7]. Microbes can synthesize a variety of volatile substances, called microbial volatile organic compounds (MVOC). The MVOC emitted by *B. amyloliquefaciens* cannot only promote the growth of *Arabidopsis*, but also activate a systemic resistance in *Arabidopsis* mediated by the ethylene- (ET) signaling pathway [8]. *Bacillus* can produce a variety of microbial plant biostimulants (MPB) to trigger or increase plant defense response, promote plant growth and improve their response to different stress [9]. The induction of systemic resistance (ISR) is a main factor involved in the suppression of plant pathogens by plant growth promoting rhizobacteria (PGPR) [10]. The secondary metabolites of *B. amyloliquefaciens* FZB42 cannot only protect plants from pathogens, but the most critical biocontrol mechanism is that the sub-lethal concentration of cyclic lipopeptides and volatiles produced by FZB42 trigger the ISR to protect plants from pathogenic microorganisms, viruses, and nematodes, and partly compensate for the changes in community structure caused by pathogens [10]. ISR is defined as the improved defensive capacity of the whole plant against a kind of broad spectrum of pathogens, acquired upon local induction by beneficial microbes [11]. Recently, molecular mechanisms of plant defense induced by PGPR have been investigated. *Arabidopsis* treated with *Bacillus cereus* AR156 shows enhanced disease resistance through the activation of systemic acquired resistance [12]. Generally, ISR depends on the lipoxygenase pathway and the jasmonic acid (JA) and/or ethylene (ET) signaling pathways against pathogens in plant hormones [13]. Unlike ISR, the system-acquired resistance is effective against pathogens dependent on salicylic acid (SA) and involves the production of pathogenesis-related proteins (PRs) not found in ISR [14]. PGPR activate the SA-signaling pathway that depends on the upregulation of the PR-1 gene [15]. By planting roots, *Bacillus* is generally able to effectively induce plant systemic resistance. Against *Botrytis cinerea*, a variety of *Bacillus* species have the ability to pre-activate systemic resistance through different mechanisms [16].

Plants are constantly exposed to multiple and complex stresses, such as pathogen attacks, cold and drought, insect bites, and so on. They have evolved a broad range of active immune mechanisms that can respond to the variety of attacks they encounter. Many studies have shown transcription factors (TFs), such as *WRKY* [17], *NAC* [18], *MYB* [19], and ET responsive factors [20], are master regulators that play the most important roles in the transcription reprogramming of plants in response to biotic or abiotic stress. Receptor-like kinases (RLKs) also play a vital role in cellular signal perception and propagation. Leucine-rich repeat RLKs are the largest group of RLKs, and are one of the largest protein super-families in plants. They play crucial roles in development and stress response [21,22]. In addition, certain defense proteins and peroxidase are involved in immune responses.

RT-PCR and microarray technology are reliable methods for examining PGPR to induce plant resistance. The complete genome of the tomato has been sequenced by an international consortium, which has accelerated phenotype screening for researchers and breeders [23]. RNA-Seq has been developed to analyze transcriptomes with or without genomic information. It is an efficient tool, promising simultaneous estimation of transcript abundance and new transcript discovery [24]. However, previous studies have focused on hormone signaling pathway networks and other proteins such as TFs and RLKs that are involved in plant defense mechanisms have remained elusive. *B. amyloliquefaciens* SN16-1 was isolated by our laboratory and can act synergistically with soil microbes to effectively control plant diseases and does not have a persistent effect on the bacterial community of tomato rhizosphere [25,26]. Here, we used the tomato as the host plant and employed the high-throughput RNA-sequencing (RNA-Seq) technology to study the gene expression and defense signaling pathways involved in SN16-1-induced resistance of tomato plants to *R. solani* RS520. The purpose of this study is to explore the mechanism of the biocontrol strain *B. amyloliquefaciens* SN16-1 in inducing tomato resistance to *R. solani*, and to provide information for the application of SN16-1 and the development of tomato varieties.

## 2. Results

### 2.1. Mapped Reads and Annotated Genes

The total RNA from the 12 samples were paired-end sequenced using the Illumina sequencing technology. In total, 38.20 Gb raw reads were generated from all samples. A total of more than 522 million filtered reads of all samples were obtained, with an average proportion of greater than 98.8%. All filtered reads were mapped to the reference genome and 97.07–97.69% uniquely-mapped reads were annotated in all samples (Table 1). The correlated coefficients between all samples ranged from 0.72 to 0.98, and replicates with low correlation coefficients were removed from further analyses.

### 2.2. Differentially Expressed Genes (DEGs) Analyses

**Figure 1:**
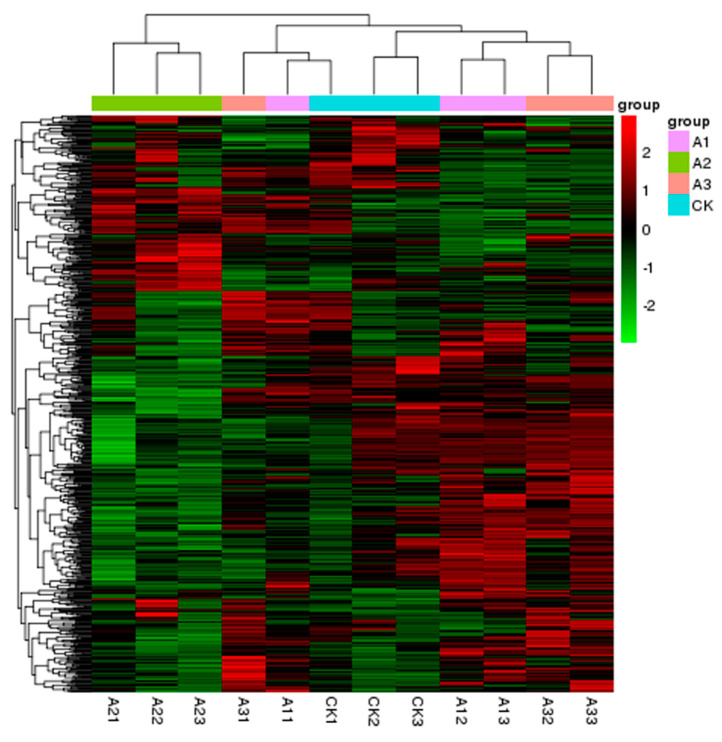
Hierarchical clustering of DEGs in tomato. Group A1 (A11, A12, A13) and A2 (A21, A22, A23) represent two groups inoculated with SN16-1 and RS520, respectively. A3 (A31, A32, A33) represents the group inoculated with SN16-1 and RS520.

DEGs from all the samples were identified by comparing the control and treatment groups, which helped give insight into the mechanism of SN16-1-induced resistance to damping-off. DEGs were identified using the package DESeq R [27]. First, the gene expression level was estimated using RPKM values (RPKM > 1). Then, DEGs were identified using the parameters (|fold change| > 2, *p*-value < 0.05). All of the analyses in this study were done using the control (CK) as a reference. In all, 130, 199, and 98 DEGs were identified in the groups inoculated with SN16-1, RS520 and SN16-1 + RS520 simultaneously, respectively (Table 2 and Table S1). The results showed that the number of DEGs did not increase in the simultaneous presence of SN16-1 and RS520. In the SN116-1 group, 19% of DEGs were related with metabolism and 47% were predicted to be related with resistance. In the RS520 group, 13% of DEGs were related to metabolism and 46% to resistance. In the SN16-1 + RS520, 7% of DEGs were involved in metabolism and 56% were related to resistance. Other genes were categorized as unknown, transport and photosynthesis. The distribution of DEGs distributions among all samples was analyzed using hierarchical clustering to gain a global view (Figure 1). The group inoculated with SN16-1 and the group inoculated with SN16-1 + RS520 had a similar distribution, which showed the gene expression patterns of the two groups were more similar to each other than they were to that of the group inoculated with RS520. In addition, there were 79 upregulated and 51 downregulated DEGs in SN16-1 group, 45 upregulated and 154 downregulated DEGs in the RS520 group and 68 upregulated and 30 downregulated DEGs in the SN16-1 + RS520 group (Table 2). The results showed that most DEGs were downregulated in the RS520 group, whereas most DEGs were upregulated in the SN16-1 and SN16-1 + RS520 groups. The expression levels of genes in three treatments were compared to those in the control at 72 hours post infection (hpi) and 130, 199, and 98 DEGs were identified in the three groups in this study. Most were upregulated in the SN16-1 and SN16-1 + RS520 groups, whereas most were downregulated in the RS520 group. After 72 h, RS520 actively suppressed tomato immunity and SN16-1 significantly improved the possible defense priming reaction. Cluster analysis found the two groups inoculated with SN16-1 and SN16-1 + RS520 together, which indicated that the gene expression was more similar in these two groups.

### 2.3. Functional Annotation of DEGs

**Figure 2:**
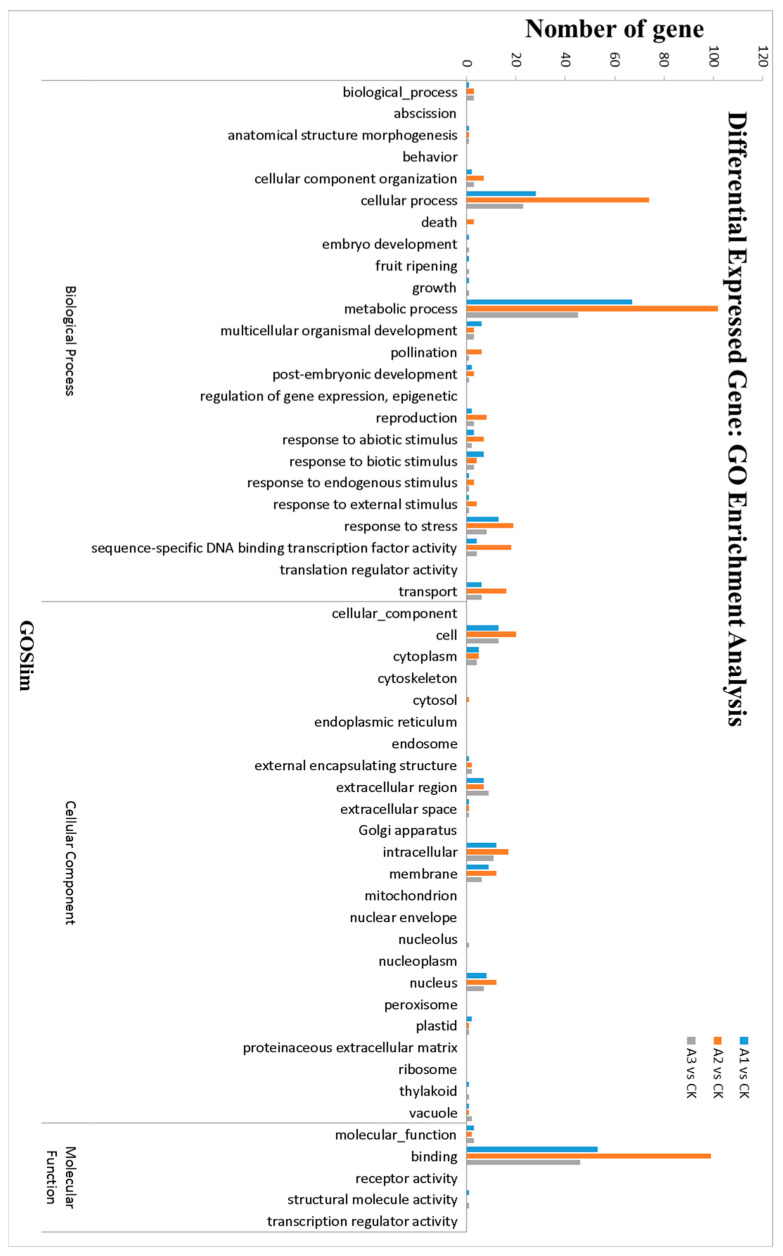
GO function enrichment analyses of DEGs.

The unigenes were classified into different functional categories based on GO annotations. The GO analyses included three major categories: cell component (CC), molecular function (MF), and biological process (BP). The GO analyses of all DEGs were performed, and the gene categories are shown in Figure 2. In the BP category, the terms cellular process, metabolic process, and response to stress were predominant. The terms of cell, intracellular and membrane were over-represented in the CC category. In MF, binding was the enriched term. The results showed that the GO categories were similar in each treatment and the numbers of gene enriched terms were different. Next, we focused on the significantly enriched GO terms (*p* < 0.05) (Table S2). In the SN16-1 group, GO:0055114, the oxidation-reduction process, GO:0061134, peptidase regulator activity, and GO:0016491, oxidoreductase activity was significantly enriched and most related DEGs were upregulated. These DEGs included peroxidase, 1-aminocyclopropane-1-carboxylate (ACC) oxidase, cytochrome P450, laccase, fatty acid desaturase, oxygenase, and so forth. The terms GO:0008037, cell recognition; GO:0010120, process of camalexin biosynthesis; and GO:0009700, the biosynthetic process of indole phytoalexin were downregulated in RS520 group. These terms included RLKs, TFs (*WRKY*, *MYB*, *NAC*, and *GRAS*) and mitogen-activated protein kinase. Most DEGs in the SN16-1 + RS520 group were related to GO:0005576, the extracellular region and GO:0048046, apoplast, which were upregulated. These genes were involved in laccase, xyloglucan endotransglucosylase, peroxidase, and germin-like protein. The significantly enriched GO terms for each treatment were different, and above all, the DEGs in the GO terms might be involved in defense.

To better understand the molecular mechanisms of the response of tomato to SN16-1 and RS520, the KEGG pathways (*p* < 0.05) that significantly respond to biotic stress were identified in all pairwise comparisons. KEGG pathway analysis showed that phenylpropanoid biosynthesis (ko00940), biosynthesis of unsaturated fatty acids (ko01040), and aldosterone synthesis and secretion (ko04925) were enriched in the SN16-1 group and most of their DEGs were upregulated. In the RS520 group, plant–pathogen interaction (ko04626), aldosterone-regulated sodium reabsorption (ko04960), the tumor necrosis (TNF) signaling pathway (ko04668) and the mitogen-activated protein kinase signaling pathway (ko04013) were significantly enriched and most DEGs were downregulated. In the SN16-1 + RS520 group, nitrogen metabolism (ko00910) was significantly enriched and many DEGs were upregulated (Table S3).

The signal transduction pathway of the plant hormones was not significantly enriched. Because the signal transduction of plant hormones plays an important role in immunity, this pathway should be focused on. The auxin and SA signaling pathways were induced in the SN16-1 group. There were no DEGs enriched in the SN16-1 + RS520. In RS520 group, the JA signaling pathway was suppressed (Table S3).

### 2.4. Differential Expression of TFs

**Figure 3:**
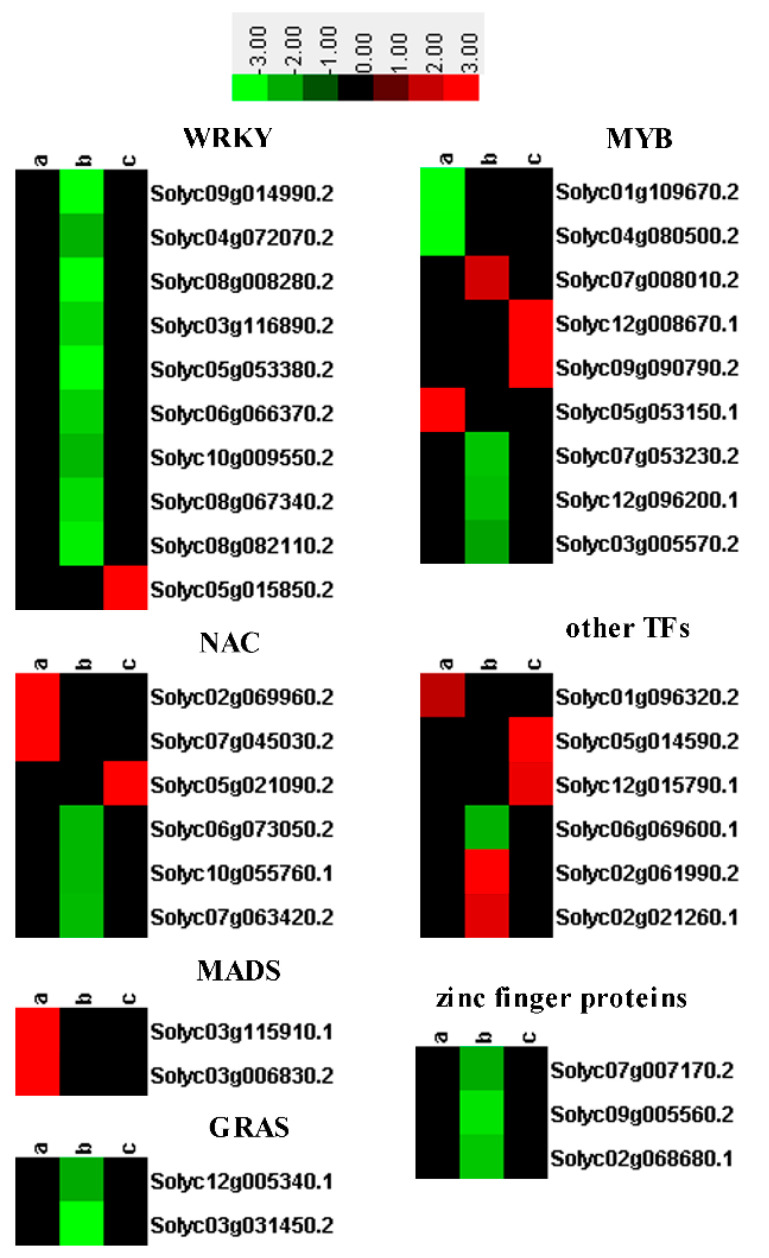
Heatmaps of DEGs encoding transcription factors. The log2 (fold change) is indicated by the colored bar using Cluster 3.0 (i.e., red for upregulated, green for downregulated), each horizontal row represents a DEGs with its gene ID and CK as reference, a, b, and c vertical columns represent SN16-1, RS520, and SN16-1+RS520.

TFs are master regulators and play important roles in the transcription reprogramming of plants in response to biotic or abiotic stress. In this study, *MYB*, *WRKY*, *NAC*, *MADS*, zinc finger protein, basic helix-loop-helix, *GRAS*, and other TFs were differentially expressed. Most TFs were upregulated in the SN16-1 and SN16-1 + RS520 groups, and most TFs were downregulated in the RS520 group (Table S1 and Figure 3).

### 2.5. DEGs Involved in Photosynthesis

**Figure 4:**
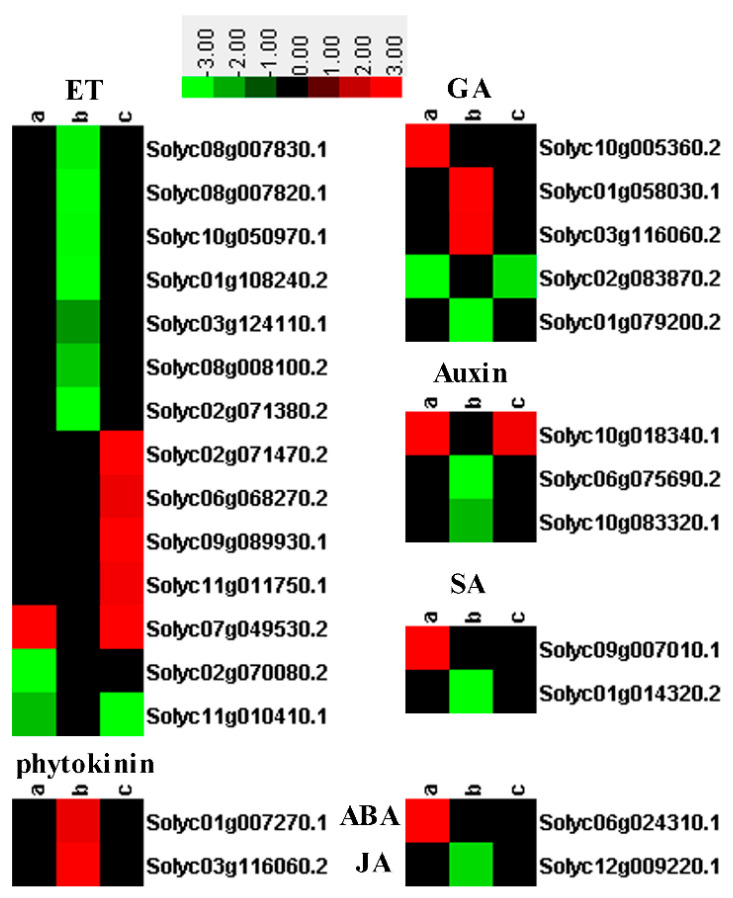
Heatmaps of DEGs involved in phytohormone, including JA, SA, ET, auxin and ABA. The log2 (fold change) is indicated by the colored bar using Cluster 3.0 (i.e., red for upregulated, green for downregulated), each horizontal row represents a DEGs with its gene ID and CK as reference, a, b and c vertical columns represent SN16-1, RS520, and SN16-1+RS520.

Phytohormones play critical roles in plant growth and resistance. Four DEGs related to plant hormones including abscisic acid (ABA), gibberellin (GA) and auxin were found in SN16-1, and three of them were upregulated. In the SN16-1 + RS520 group, three DEGs including ET and auxin were upregulated and one, GA, was downregulated. In the RS520 group, three DEGs encoding GA were upregulated and eight including ET, SA, and JA, were downregulated (Table S1 and Figure 4).

### 2.6. Differential Expression of RLKs and Heat Shock Proteins

**Figure 5:**
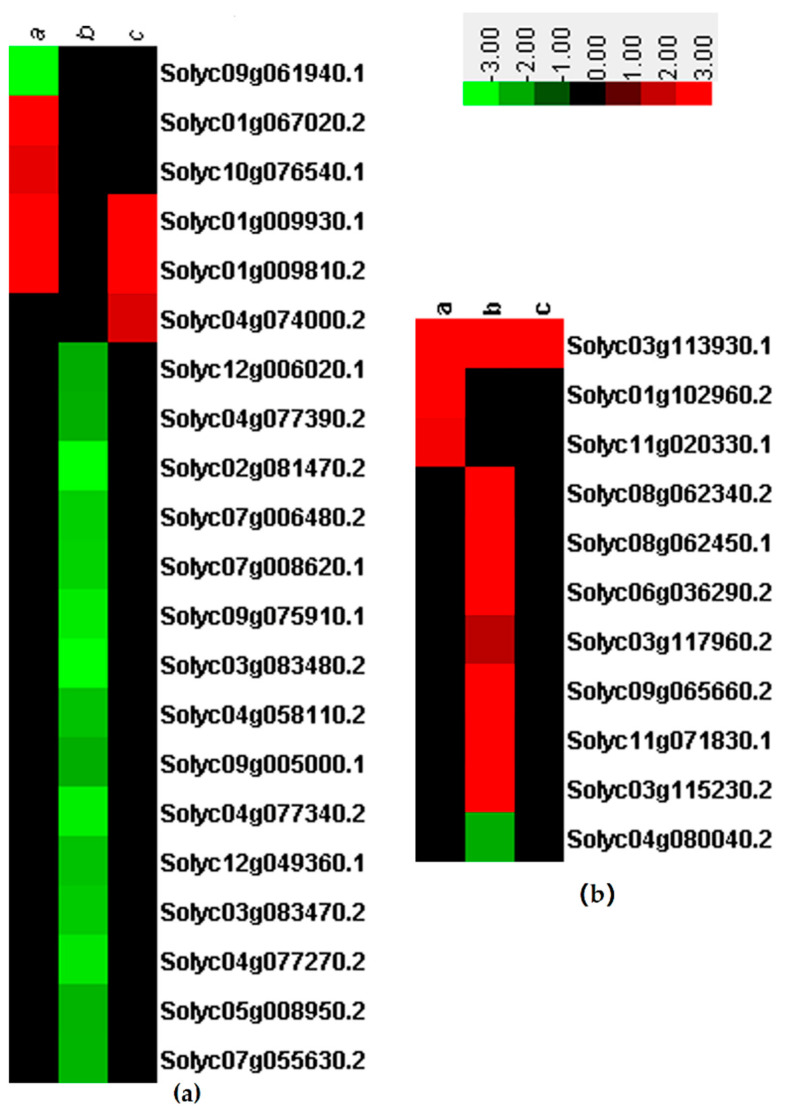
The log2 (fold change) is indicated by the colored bar using Cluster 3.0 (i.e., red for upregulated, green for downregulated), each horizontal row represents a DEGs with its gene ID and CK as reference, a, b and c vertical columns represent SN16-1, RS520, and SN16-1+RS520. (**a**) Heatmaps of DEGs involved in RLKs. (**b**) Heatmaps of DEGs involved in HSPs.

RLKs in plants are known to function in diverse biological processes including growth and development, hormone perception and plant–microbe interactions. In our experiments, five DEGs were found to be related to RLKs and four of them were upregulated in the SN16-1 group. In the SN16-1 + RS520 group, three DEGs were upregulated and one was downregulated. In the RS520 group, all 19 DEGs were downregulated (Table S1 and Figure 5A). Heat shock proteins (HSPs) constitute a stress-responsive family of proteins that are essential for maintaining cellular homeostasis under stressful conditions in plants. We found that most HSPs were downregulated in the RS520 group, including HSP20 and HSP90, and HSPs were upregulated in the SN16-1 and SN16-1+RS520 groups (Figure 5B).

### 2.7. DEGs Involved in Resistance

DEGs, such as proteinase inhibitor, chitinase, peroxidase, u-box domain-containing protein, lateral organ boundaries (LOB) domain protein, beta-1 3-glucanase, cytochrome P450, calmodulin, PRs, and the nucleotide-binding site (NBS-LRR) resistance protein, were involved in resistance. Detailed results are displayed in Table 3 and Table S1. Most DEGs were upregulated in presence of SN16-1 + RS520 and were downregulated in presence of RS520. However, PRs were seldomly expressed.

### 2.8. Validation of Differentially Expressed Transcripts from RNA-Seq

**Figure 6:**
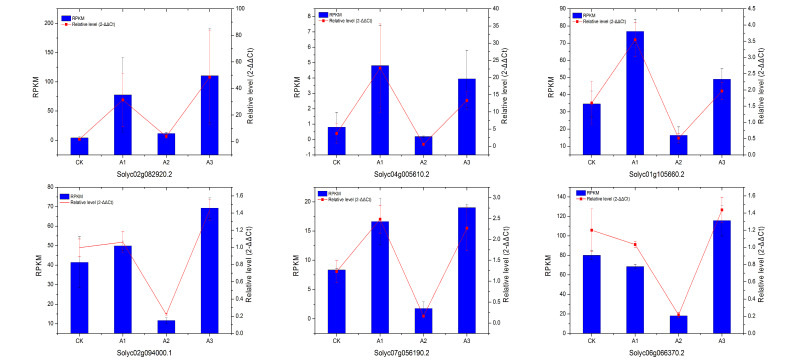
The relative expression level change of 6 selected genes from DEGs by quantitative real-time PCR. Left vertical coordinate is RPKM of RNA-Seq; right vertical coordinate is relative expression level of qRT-PCR.

Six genes coded *WRKY33*, *CML*, cytochrome P450, ACC, PR, and endochitinase were randomly selected to confirm the expression patterns in the RNA-Seq results using real-time qPCR (Figure 6). The results showed a similar change trend for all tested unigenes, which implied a high degree of reliability for the RNA-Seq data.

## 3. Discussion

In the recent years, *B. amyloliquefaciens* has been developed as biological control agent to control plant pathogens. Many studies have showed that *B. amyloliquefaciens* plays a vital role in inducing systemic resistance in plants to pathogens [10,11]. However, few studies have examined the molecular mechanism of induced plant resistance against pathogens. In the present study, RNA-Seq was employed to investigate the changes in gene expression in tomato plants after inoculation with *B. amyloliquefaciens* SN16-1 and RS520.

KEGG analysis showed that phenylpropanoid biosynthesis was significantly upregulated in the SN16-1group. The secondary metabolites, phenylpropanoids, and their derivatives, relevant to plant survival, have been elucidated in *Arabidosis* and other species [28]. In addition, the auxin signaling pathway (small auxin upregulated RNA, SAUR, Solyc10g018340.1) and the SA signaling pathway (PR-1, Solyc09g007010.1) were also induced in SN16-1. SAUR is largest family of early auxin response genes, which are the key effector outputs of hormonal and environmental signals that regulate plant growth and development [29]. SA is a critical signaling molecule that mediates plant defense responses against numerous biotrophic/hemibiotrophic pathogens, as well as ISR, and it confers a long-lasting, broad spectrum resistance against pathogen infection [14]. The results showed that SN16-1 not only promoted plant growth, but also activated plant immunity via SA signaling. Generally, PGPR induce plant resistance via JA- and/or ET- signaling pathways. Interestingly, in this study, tomato plants treated with SN16-1 had increased levels of SA. It was previously reported that the *B. cereus* AR156 induced *Arabidopsis* SAR through the SA signal pathway to enhance its disease resistance [12]. In addition, certain genes related to ABA and ET were also differentially expressed in the SN16-1 group. Abscisic acid insensitive 8 (Solyc06g024310.1) was upregulated in SN16-1, which encodes a novel protein mediating ABA [30]. ABA has been repeatedly shown to paralyze plant defenses by antagonizing the SA pathway in *Arabidopsis*, but this antagonism can contribute significantly to immunity as well as to responses to environmental stresses [31]. ACC oxidases were also differentially expressed in SN16-1. ACC is the precursor of ET, which is oxidized by ACC oxidase to form it. It is a stress hormone that modulates a diverse array of the defense response [32,33]. In RS520, JA signaling (JAZ, Solyc12g009220.1) was downregulated. The JA played an important role in tomato resistance to RS520. In addition, all genes encoding ET-responsive TFs and ACC oxidases were downregulated, which implies that ET hormones were suppressed by RS520. However, most genes involved in ET hormones were upregulated in SN16-1 + RS520. ET-responsive TFs play critical roles in plant immunity [34,35]. In *Arabidopsis*, ET-responsive factors have been shown to act as regulators of the JA/ET signaling pathway [36,37] and they also had the same function in tomato plants [38]. In our experiment, ET-responsive factors may have played vital role against RS520.

In the RS520 group, plant–pathogen interaction was significantly downregulated. These included genes such as TFs *WRKY25* (Solyc06g066370.2), *WRKY33* (Solyc09g014990.2), and Calmodulin-like (*CML*, Solyc02g094000.1). Previous studies have shown that *WRKY25* and *WRKY33* play vital roles in responses to pathogen attacks [39,40,41]. CML proteins are primary Ca2+ sensors that control diverse cellular functions, such as mediating signaling responses in defense against various pathogens [41]. The downregulation of *WRKY25*, *WRKY33*, and *CML* in tomato might promote their infection by RS520.

TFs including *WRKY*, *MYB*, *NAC*, MADS-box zinc finger protein and *GRAS* were differentially expressed, which are regulators of plant defense response and development [18,42,43,44,45,46]. MADS-box genes function in endothelium development [47], root development [48], and sepal and fruit formation [49]. They were expressed only in the SN16-1 group, which indicated that MADS-box genes might be related to development and have nothing to do with defense. NAC TFs were induced in presence of SN16-1, while they were repressed in presence of RS520. An increasing body of evidence implies that NAC genes regulate defense networks to respond to pathogen infection and environment stimuli [50,51]. It is thought that NAC genes positively regulate defense response by activating the PR gene, inducing a hypersensitive response and cell death at the site of infection, or negatively regulate plant basal pathogen resistance by suppressing PR1 expression [52,53,54,55]. The PR gene might be involved in the defense of tomato plant against RS520. It is well documented that *MYB* genes are involved in a variety of functions, such as abiotic stress responses [56] and anthocyanin [57]. However, there have been few studies on the response of biotic stress. In this study, *MYB* genes were either up- or downregulated in all three treatments, which illustrates that the gene might have a role in the response to biotic stress. Notably, all WRKYs were downregulated in the RS520 group, including *WRKY33* and *WRKY25*. Only one *WRKY* gene was upregulated in the SAN16-1 + RS520 group, and no gene was differentially expressed in the SN16-1 group. The result suggests that *WRKY* genes in the tomato might be the most susceptible to pathogens and play an important role in response to RS520.

Multiple RLKs genes were differentially expressed in treatment groups. They play a vital role in perception of extracellular stimuli and the activation of downstream processes [22]. DEGs involved in LRR RLKs were upregulated in the SN16-1 and SN16-1 + RS520 groups and were downregulated in the RS520. This result suggests LRR-RLK genes might reflect a defense priming response in our experiment.

It is well known that HSPs are a subset of molecular chaperones that are rapidly induced in large numbers by stress [58]. Our results showed that HSP20s exhibited a range of responses to SN16-1 and RS520. Research has shown that HSP20s can be profusely induced by in tomato plants by biotic stressors such as *B. cinerea* and the tomato wilt virus and the HSP20s gene family plays an importance role in pathogenesis [59].

Many defense proteins, such as peroxidase, beta-1, 3-glucanase, proteinase inhibitor and LRR resistance protein, were upregulated in SN16-1 + RS520 and downregulated in RS520. In SN16-1, regulated proteins were differentially expressed, including the LOB domain protein and u-box domain-containing protein. These results implied that SN16-1 might only induce regulatory proteins and rarely induce PRs. PGPR seems to prepare the responses to pathogens by priming the plant to respond more rapidly rather than activating a constitutive defense [60].

In conclusion, our results show that SN16-1 can effectively induce tomato resistance to *R. solani*. SN16-1 induces plant disease resistance by inducing tomato transcription factors (MYB, NAC and MADS), plant hormones (ethylene, auxin, abscisic acid, and gibberellin), RLKs, HSPs and defense proteins. SN16-1 activates the SA signal by inducing the upregulation of PR1, and when pathogens are present at the same time, the plant’s defense protein is activated, indicating that SN16-1 alone does not activate the plant’s defense system, but when pathogens attack, SN16- 1 can prompt plants to quickly activate the defense system and realize plant disease resistance.

## 4. Materials and Methods

### 4.1. Bacterial and Fungal Strains

The *B. amyloliquefaciens* SN16-1 was preserved in our laboratory, and grown in Luria Broth (LB) medium at 30 °C, 200 r/min for 12 h. After centrifugation at 12,000× *g* for 10 min, the precipitate was suspended in distilled water. The bacterial counts were performed with standard plate counts. The pathogen RS520 was cultured in Potato Dextrose Agar (PDA) medium at 28 °C for 6 d. The hyphae collected from the plate were suspended in sterile water and inoculated by root irrigation.

### 4.2. Plant Materials and Treatment

A pot experiment was carried out to determine the disease-resistant genes of tomato plants in a PGPR and pathogen inducing condition. Tomato seeds (Fen Fan) were placed in 75% alcohol for 30 s, and then transferred into 10% Nalco for 10 min to kill most surface microorganisms. Finally, the seeds were rinsed three times in sterile water. Then, they were transferred to pots filled with 500 g dry soil and cultured in the illumination incubator. The temperature was set to 28 °C, the illumination time was 14 h/d, and humidity was maintained at 60%. Upon the appearance four leaves, strains of SN16-1 and *R. solani* (RS520) were used to artificially inoculate the tomato. The SN16-1 was activated twice in LB solid medium at 37 °C for 12 h and then inoculated into LB liquid medium at 30 °C, 200 r/min for 10 h. The strains were collected by centrifuging and re-suspended in sterile water. The strain RS520 was incubated in potato dextrose agar solid medium at 28 °C for 6 d.

At the four-leaf stage of tomato, three treatments of artificial inoculation were carried out. SN16-1 (3.34 × 10^8^ cfu/g dry soil) were inoculated by root-irrigation; RS520 (2.14 g/g dry soil) was inoculated in the tomato root; the SN16-1 and RS520 were inoculated simultaneously. The control group was watered with water alone. Each treatment had three repetitions. After culturing for 72 h, we collected similarly sized leaves from each group. The samples were stored at −80 °C after being frozen in liquid nitrogen in preparation for the next stage.

### 4.3. RNA Extraction, mRNA-Seq Library Construction and Sequencing 

Four leaf samples were pooled together for RNA isolation. The RNA of the samples was extracted using TRIzol Reagent (Invitrogen) according to the manufacturer’s protocol. mRNA was purified from the total RNA (0.75 μg) using magnetic beads with oligo-dT to combine with mRNA poly-A tail. Then, the mRNA was mixed with a fragmentation buffer to obtain a short fragment of 200–300 bp. These fragments were used to synthesize cDNA with random primers, RNase H, and DNA polymerase I. The cDNAs were purified using a QIAquick PCR Extraction Kit (Qiagen, Valencia, CA, USA) and then end-repaired. The end-repaired DNA fragments were ligated with sequencing adapters through A and T complementary base pairing. The multiplexed cDNA libraries were checked using PicoGreen and fluorospectrophotometry and quantified using an Agilent 2100 Bioanalyzer. The sequencing library was gradually diluted and quantified to 4–5 pM and sequenced using the Illumina NextSeq™ 500 platform (San Diego, CA, USA).

### 4.4. Transcriptome Data Processing and Analysis

RNA-Seq-filtered reads were obtained by removing low-quality reads (Q < 20) from raw data. The remaining high-quality pair-end reads for each sample were mapped to the tomato genome sequence published by the Tomato Genome Consortium [25] using TopHat [61]. All transcripts were assembled using the program Cufflinks. The gene model annotation was derived from the Ensemble database (http://www.ensemble.org/; accessed on 12 December 2016). The RPKM of each gene was calculated to estimate gene expression levels, which are based on the length of the gene and the read count mapped to it [62]. Differential expression analysis was performed by the program DEGSeq. The DEGs between samples were selected using the following criteria: (i) the logarithmic of fold change < 2; (ii) the false discovery rate FDR-corrected *p*-values < 0.05 (using Benjamini–Hochberg adjustment). The Gene Ontology (GO) terms and Kyoto Encyclopedia of Genes and Genomes (KEGG) enrichment analysis of DEGs were implemented according to previous methods [63,64]. All primary sequencing data were deposited in the National Center for Biotechnology Information Gene Expression Omnibus (GEO) database under accession number SPR103275.

### 4.5. qRT-PCR Analysis

The qRT-PCR was employed to analyze a subset of the transcripts (for each sample, three biological replicates). RevertAid First Strand cDNA Synthesize Kit (Thermo Scientific Fermentas, Darmstadt, Germany) was used to reverse-transcribe the RNA of all samples into cDNA, according to the instructions. Six DEGs involved in defense pathways were selected. The software primer5 was used to design gene specific primers. qPCR was performed using ITaq universal SYBR green supermix (Bio-Rad, Hercules, CA, USA) using the following protocol: 95 °C for 20 s, 40 cycles of 95 °C for 3 s, and 60 °C for 30 s, followed by a melt curve analysis. The results of the qRT-PCR reactions were analyzed using the comparative cycle threshold (CT) method (ΔΔCT), and the mock-treated recurrent parent was used as the calibrator. The data analysis was performed with the 2^−ΔΔCt^ method. The qRT-PCR experiments were performed in biological triplicate, with three technical replicates. The genes selected for validation and primer sequences are listed in Table S4.

## Figures and Tables

**Table 1:** Numbers of RNA-Seq reads generated for each sample that was filtered and mapped events count.

Sample ^1^	Total Reads No.	Clean Reads		Total Mapped		Uniquely Mapped	
		Reads No.	%	Reads	%	Reads	%
A11	41591936	41115060	98.85	38156224	92.8	37826715	99.14
A12	46933954	46356470	98.76	41760720	90.09	41408274	99.16
A13	45159328	44603996	98.77	40450372	90.69	40134671	99.22
A21	42489140	42034172	98.92	38951058	92.67	38468456	98.76
A22	43035854	42536132	98.83	38671015	90.91	38363246	99.2
A23	44400866	43889800	98.84	40142535	91.46	39865840	99.31
A31	43185936	42721764	98.92	37825546	88.54	37495403	99.13
A32	47183870	46627944	98.82	42188529	90.48	41875921	99.26
A33	43779638	43279510	98.85	39113518	90.37	38819363	99.25
CK1	43424342	42940222	98.88	38632864	89.97	38266786	99.05
CK2	43252592	42741110	98.81	38992852	91.23	38682597	99.2
CK3	43935896	43417912	98.82	39554495	91.1	39238937	99.2

^1^ CK (CK 1, CK 2, CK 3) represents the control. Group A1 (A11, A12, A13) and A2 (A21, A22, A23) represent two groups inoculated with SN16-1 and *R. solani* (RS520), respectively. A3 (A31, A32, A33) represents the group inoculated with SN16-1 and *R. solani* (RS520).

**Table 2:** Number of DEGs identified in strains-treated compared with mock-treated tomato leaves.

Case	Control	Upregulated Genes	Downregulated Genes	Total DEGs
SN16-1	CK	79	51	130
RS520	CK	45	154	199
SN16-1+RS520	CK	68	30	98

**Table 3:** DEGs related to resistance in strains-treated compared with mock-treated tomato leaf.

ID	Fold-Change			Description
	a ^1^	b ^1^	c ^1^	
Solyc03g020010.1	0	0	12.09	Kunitz-type trypsin inhibitor
Solyc03g020050.2	0	0	32.75	Proteinase inhibitor II
Solyc07g054720.1	0	0	214.09	Proteinase inhibitor type-2
Solyc09g089510.2	0	−16.71	0	Proteinase inhibitor I
Solyc09g084490.2	0	−20.05	0	Proteinase inhibitor I
Solyc09g084440.2	29.62	0	0	Proteinase inhibitor I
Solyc09g083440.2	9.65	0	0	Proteinase inhibitor I
Solyc02g082920.2	15.16	0	21.65	Chitinase
Solyc07g005100.2	0	0	9.04	Chitinase-like protein
Solyc01g008620.2	0	0	19.57	Beta-1, 3-glucanase
Solyc02g086700.2	0	0	85.36	Beta-1, 3-glucanase
Solyc01g059980.2	0	0	6.3	Beta-glucanase
Solyc02g087070.2	0	0	44.32	Peroxidase family protein
Solyc06g050440.2	0	0	6.36	Peroxidase
Solyc09g072700.2	0	0	6.28	Peroxidase 57
Solyc02g064970.2	0	36.56	0	Peroxidase
Solyc03g006810.2	0	3.56	0	Peroxidase
Solyc08g080590.2	0	2.3	0	Thaumatin pathogenesis-related protein
Solyc02g087520.2	0	2.07	0	Thaumatin-like protein
Solyc10g075150.1	0	0	66.15	Non-specific lipid-transfer protein
Solyc08g007460.2	0	0	11.2	Non-specific lipid-transfer protein
Solyc04g007320.1	0	−4.69	0	TIR-NBS-lRR resistance protein
Solyc02g090380.2	0	−4.86	0	NBS-LRR resistance protein
Solyc01g102840.2	0	−5.89	0	TIR-NBS resistance protein
Solyc07g056190.2	0	−7.71	0	NBS-LRR class R gene resistance protein
Solyc02g084890.1	0	−4.18	0	CC-NBS-lRR resistance protein
Solyc05g013250.1	0	0	14.40	LRR resistance protein
Solyc09g091210.2	0	−6.65	0	R gene resistance response
Solyc04g064870.2	0	−4.25	0	Pathogenesis-related protein-like protein
Solyc02g094000.1	0	−4.35	0	Calmodulin-like protein
Solyc03g113980.2	0	−2.52	0	Calmodulin-binding protein
Solyc03g119250.2	0	−5.75	0	Calmodulin-binding protein
Solyc11g072470.1	5.13	0	0	LOB domain protein 1
Solyc03g112430.1	17.04	4.46	0	LOB domain protein 1
Solyc05g046290.2	0	0	11.11	Xyloglucan hydrolase 2
Solyc11g068940.1	38.63	0	0	U-box domain-containing protein 24
Solyc11g006030.1	9.49	0	0	U-box domain-containing protein
Solyc08g077900.2	326.16	0	465.02	Expansin-like protein
Solyc07g055690.1	0	0	67.6	S-locus-specific glycoprotein S6
Solyc07g062480.1	0	0	14.62	S-locus glycoprotein
Solyc08g079900.1	0	0	22.26	Subtilisin-like protease

^1^ a, b, and c represent SN16-1, RS520, and SN16-1+RS520, respectively.

## Data Availability

The data presented in this study are openly available in the National Center for Biotechnology Information Gene Expression Omnibus (GEO) database under accession number SPR103275 and Supplementary Material of this article.

## Supplementary Materials

The following are available online at https://www.mdpi.com/article/10.3390/pathogens11010035/s1, Table S1: Differentially expressed genes between treatment and control, Table S2: GO enrichment of differentially expressed genes between treatment and control, Table S3: KEGG pathway of differentially expressed genes between treatment and control, Table S4: Sequence of forward and reverse primers used in Real time RT-PCR experiments.
